# Dynamics trajectory of patient-reported quality of life and its associated risk factors among hepatocellular carcinoma patients receiving immune checkpoint inhibitors: a prospective cohort study

**DOI:** 10.3389/fimmu.2024.1463655

**Published:** 2024-11-04

**Authors:** Xue-Mei You, Fei-Chen Lu, Fan-Rong Li, Feng-Juan Zhao, Rong-Rui Huo

**Affiliations:** ^1^ Hepatobiliary Surgery Department, Guangxi Medical University Cancer Hospital, Nanning, China; ^2^ Key Laboratory of Early Prevention and Treatment for Regional High Frequency Tumor (Guangxi Medical University), Ministry of Education, Nanning, China; ^3^ Guangxi Key Laboratory of Early Prevention and Treatment for Regional High Frequency Tumor, Nanning, China; ^4^ Medical Imaging Department, Guangxi Medical University Cancer Hospital, Nanning, China; ^5^ Head and Neck Surgery Department, Guangxi Medical University Cancer Hospital, Nanning, China; ^6^ Department of Experimental Research, Guangxi Medical University Cancer Hospital, Nanning, China

**Keywords:** hepatocellular carcinoma, quality of life, trajectory analysis, immunotherapy, immune checkpoint inhibitors

## Abstract

**Objective:**

We aimed to characterize quality of life (QOL) trajectories among patients with intermediate and advanced hepatocellular carcinoma patients treated with immunotherapy.

**Methods:**

Barcelona Clinic Liver Cancer (BCLC) stage B-C HCC patients receiving immunotherapy at Guangxi Medical University Cancer Hospital were included. Trajectories of QOL, assessed using the Functional Assessment of Cancer Therapy-Hepatobiliary (FACT-Hep) questionnaire, were identified through iterative estimations of group-based trajectory models. Associations with trajectory group membership were analyzed using multivariable multinomial logistic regression.

**Results:**

Three trajectory groups were identified (n=156): excellent (35.3%), poor (43.6%), and deteriorating (21.1%) QOL. The deteriorating trajectory group reported a mean QOL score of 124.79 (95% CI, 116.58–133.00), but then declined significantly at month-2 (estimated QOL score 98.67 [95% CI, 84.33–113.00]), and the lowest mean score is reached at month-6 (estimated QOL score 16.58 [95% CI, 0–46.07]). Factors associated with membership to the deteriorating group included no drinking (odds ratio [OR] *vs* yes [95% CI], 3.70 [1.28–11.11]), no received radiotherapy (OR *vs* yes [95% CI], 8.33 [1.41–50.00]), diabetes (OR *vs* no [95% CI], 6.83 [1.57–29.73]), and extrahepatic metastasis (OR *vs* no [95% CI], 3.08 [1.07–8.87]). Factors associated with membership to the poor group also included body mass index ≤24.0 kg/m^2^ (OR vs no [95% CI], 4.49 [1.65–12.22]).

**Conclusions:**

This latent-class analysis identified a high-risk cluster of patients with severe, persistent post-immunotherapy QOL deterioration. Screening relevant patient-level characteristics may inform tailored interventions to mitigate the detrimental impact of immunotherapy and preserve QOL.

## 1 Introduction

Hepatocellular carcinoma (HCC), the most common primary liver cancer, is a significant global cause of morbidity and mortality (1, 2). Recent years have witnessed a substantial transformation in the HCC treatment landscape, particularly with an expanded array of therapeutic options for advanced disease (2–4). However, a predominant challenge remains as most HCC patients are diagnosed at an advanced stage, precluding them from curative treatments (5). The past decade has seen significant advancements in pharmacotherapy for advanced HCC (2, 5, 6). Notably, the oral small molecule multikinase inhibitors (MTKis) — sorafenib, cabozantinib, regorafenib, and lenvatinib — along with the monoclonal antibody (mAb) ramucirumab, have demonstrated effectiveness in phase III clinical trials as first- or second-line treatments (7–12). These drugs have received approval from the United States Food and Drug Administration (FDA) for use in advanced HCC. Concurrently, immunotherapies, particularly nivolumab and pembrolizumab, have shown promising outcomes, leading to their conditional FDA approval for second-line use (13, 14). Despite these advancements, which have improved overall survival (OS) rates for advanced HCC patients, these therapies are not curative. Additionally, their unique treatment-related toxicities can exacerbate patients’ already fragile health. Therefore, treatment strategies that also prioritize maintaining an adequate quality of life (QOL) are of paramount importance.

QOL encompasses complete physical, mental, and social well-being, extending beyond the mere absence of disease or infirmity. The European Society of Medical Oncology (ESMO) emphasizes the importance of QOL, incorporating it as a critical parameter in evaluating the clinical value of anticancer treatments (15, 16). Despite this recognition, QOL assessments are often overlooked or inadequately reported in phase III clinical trials (17, 18). Previous research has shown that immunotherapy generally has a transient negative impact on QOL (19, 20). However, traditional analytic methods, which describe average outcomes at the population level, may fail to capture the individual variability in the longitudinal trajectory of cancer-related QOL. But, more detailed and nuanced exploration of this variability is possible through clustering techniques, such as growth mixture models or latent class analyses. These methods can identify patient subgroups with similar longitudinal trajectories, offering a more comprehensive understanding of the impact of cancer and its treatment over time (21, 22). To our best knowledge, no studies have attempted to identify distinct groups of HCC patients who experience distinct QOL trajectories after immunotherapy using this methodological approach.

Early identification of high-risk groups for QOL deterioration is crucial for timely, patient-specific supportive care interventions. This study was conducted among intermediate and advanced HCC patients who received immunotherapy, with the following aims: (1) to describe dynamics of patient-reported QOL over six months after immunotherapy; (2) to identify patients at high risk of QOL deterioration; and (3) to focus on factors are associated with distinct patterns of QOL.

## 2 Methods

### 2.1 Study design and patients

Barcelona Clinic Liver Cancer (BCLC) stage B-C HCC patients receiving immunotherapy at Guangxi Medical University Cancer Hospital from January to July 2023 were initially included. Enrollment criteria were as follows: diagnosis of HCC confirmed by postoperative histopathology; no prior immunotherapy; age 18-75 years; Eastern Cooperative Oncology Group (ECOG) performance score of 0-1; Child-Pugh score ≤7; Karnofsky performance score (KPS) >60; estimated survival time ≥6 months; no family history or history of mental illness, no consciousness disorders, and normal cognitive function. Exclusion criteria were: presence of other malignant tumors; tumor-related surgery within the last two months; concurrent use of Chinese herbal medicines with anti-tumor effects; active or historical autoimmune systemic diseases with potential relapse; discontinuation of subsequent treatment due to severe immune-related adverse reactions (e.g., immune-related myocarditis, hepatitis, colitis).

The study protocol was approved by the Ethics Review Committee of Guangxi Medical University Cancer Hospital (KY2024397) and conformed to the Declaration of Helsinki. All enrolled patients provided informed consent prior to project initiation. This study was conducted in accordance with the Strengthening the Reporting of Observational Studies in Epidemiology (STROBE) guidelines (23).

### 2.2 Immunotherapy regimen

Treatment plans for all patients are evaluated and determined by a minimum of two attending physicians based on the patient’s condition. Medication may be discontinued in cases of disease progression or intolerable adverse reactions. The immune checkpoint inhibitors include PD-1 inhibitors such as sintilimab, toripalimab, camrelizumab, pembrolizumab, and nivolumab, all administered intravenously at a dosage of 200 mg every three weeks. Additionally, the PD-L1 inhibitor atezolizumab is administered intravenously at a dosage of 1200 mg every three weeks.

### 2.3 Outcome variable and follow-up

The primary outcome was the QOL, which was determined through the use of the Functional Assessment of Cancer Therapy-Hepatobiliary questionnaire (FACT-Hep) (24). The 45-item FACT-Hep consists of five subscales: physical well-being; social and family well-being; emotional well-being; functional well-being; and the hepatobiliary cancer subscale (HepCS). The HepCS includes 18 items that assess specific symptoms of hepatobiliary carcinoma and side-effects of its treatment. Aggregate scores can also be formed, from 0 to 180, higher scores on all scales of the FACT-Hep reflect better quality of life or fewer symptoms.

Following immunotherapy, patients undergo follow-up examinations every two months. These examinations comprise blood routine tests, liver and kidney function tests, key tumor markers, enhanced abdominal CT or MRI, and chest CT. QOL assessments occur at four intervals: baseline (prior to immunotherapy), two months post-immunotherapy, four months post-immunotherapy, and six months post-immunotherapy. The study includes patients with a minimum of two measurement OQL.

### 2.4 Variables of interest

Data collected by medical record review at diagnosis included age, sex, drinking status, income; history of family cancer, diabetes, and hypertension; hepatitis B surface antigen, hepatitis C antibody, liver cirrhosis, body mass index, tumor number, tumor size, extrahepatic metastasis, vascular invasion, a-fetoprotein, BCLC stage, targeted therapy regimens, immunotherapy regimens, liver resection, transarterial chemoembolization, and radiotherapy. Body mass index was calculated as weight in kilograms divided by height in meters squared.

### 2.5 Statistical analyses

Data for normally distributed continuous variables were presented as means and standard deviations (SDs). Categorical variables were described using frequencies and percentages. Baseline characteristics, summarized by QOL trajectory groups, were compared using the χ^2^ test or analysis of variance, as appropriate.

Longitudinal variations in the FACT-Hep Summary Score were analyzed using Group-Based Trajectory Modeling (GBTM) (21, 22, 25). This approach enabled the identification of polynomial trajectories and latent trajectory groups, which are clusters of individuals with similar outcome progressions. Model selection was meticulous, involving iterative estimations to determine the optimal fit. This included deciding on the number of trajectory groups and the shape or order of each group using maximum likelihood methods. Time was categorized in weeks for estimating trajectory groups. A detailed description of the model selection process is provided in the **Supplementary Methods**. Each identified trajectory group was assigned a descriptive label to succinctly represent its QOL outcome patterns. Following this, we characterized the demographics of participants in each group. To enhance insights from the FACT-Hep Summary Score and provide a detailed view of the dynamics of its components, mean scores across all FACT-Hep Questionnaire scales were compiled and summarized by trajectory group.

A multivariable multinomial logistic regression model was subsequently used to estimate the associations between baseline covariates and trajectory group membership, the association size was expressed as odds ratio (OR) and its 95% confidence interval (CI). The optimal pattern of the FACT-Hep Summary Score was selected as the reference point. This approach was adopted to concentrate on identifying factors associated with clustering into groups characterized by less favorable patterns.

Analyses were performed using R, v4.3.0 (R Foundation), and the GBTM model was fitted using lcmm package. Statistical significance was defined with a P value < 0.05.

## 3 Results

### 3.1 Cohort characteristics

**Figure 1:**
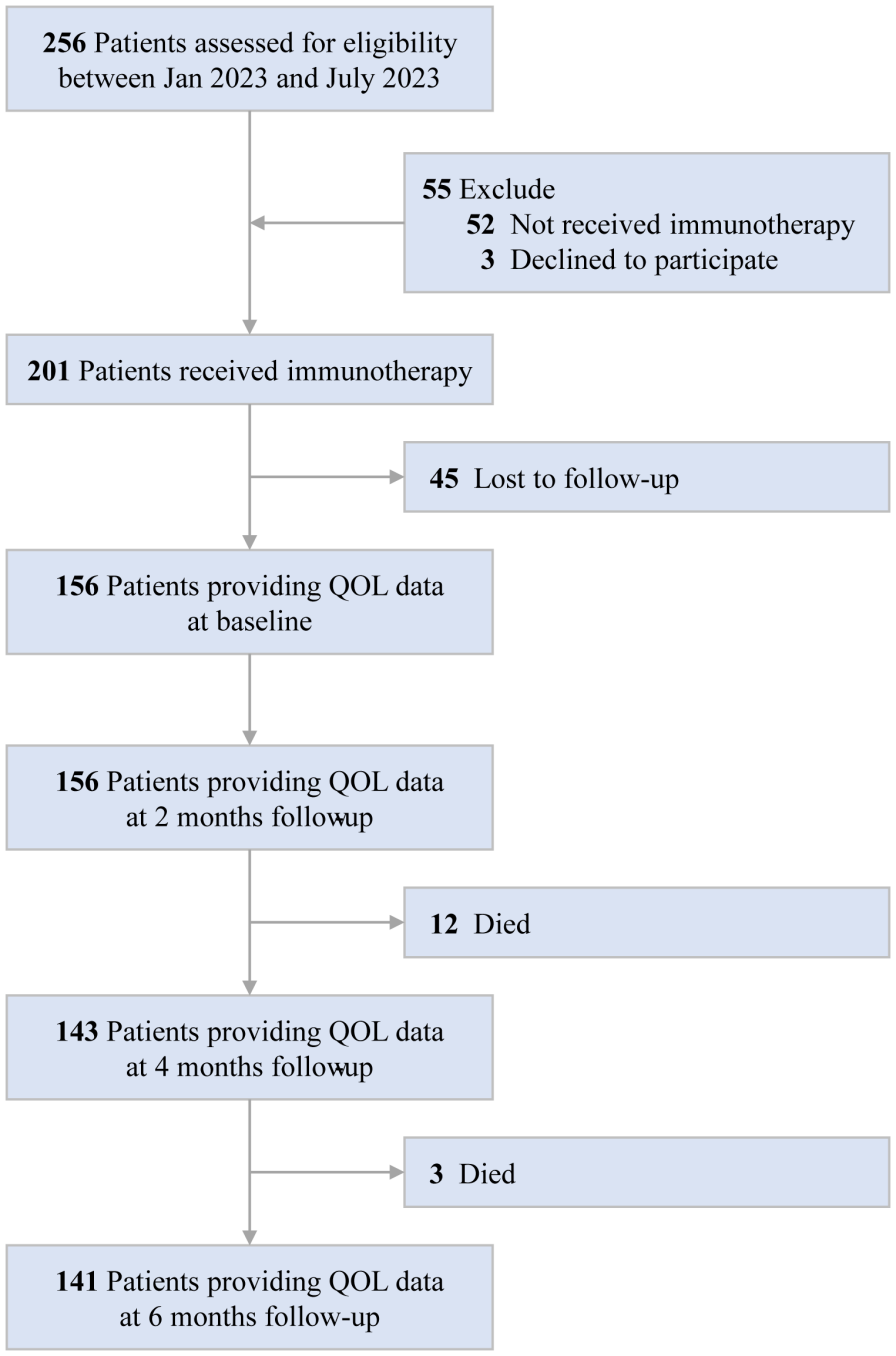
Consort diagram of patient population. QOL, quality of life.

Of the 256 HCC patients at study baseline, we excluded 52 who did not receive immunotherapy, 3 who declined to participate, and 45 who were lost to follow-up. Consequently, 156 patients were included in the analysis. A detailed description of the selection process is provided in **Figure 1**. In the whole cohort (n=156), the mean age was 50.81 years (SD 10.48), 140(89.7%) and 16(10.3%) patients were male and female, respectively; 37(23.7%) and 119(76.3%) patients were BCLC B and C stage, respectively. Overall, 67.9% received targeted therapy with Lenvatinib, 67.3% received immunotherapy with Tislelizumab, 13.5% received liver resection, 81.4% received transarterial chemoembolization therapy, and 13.5% received radiotherapy (**Supplementary Table S1**).

### 3.2 QOL trajectory groups

**Figure 2:**
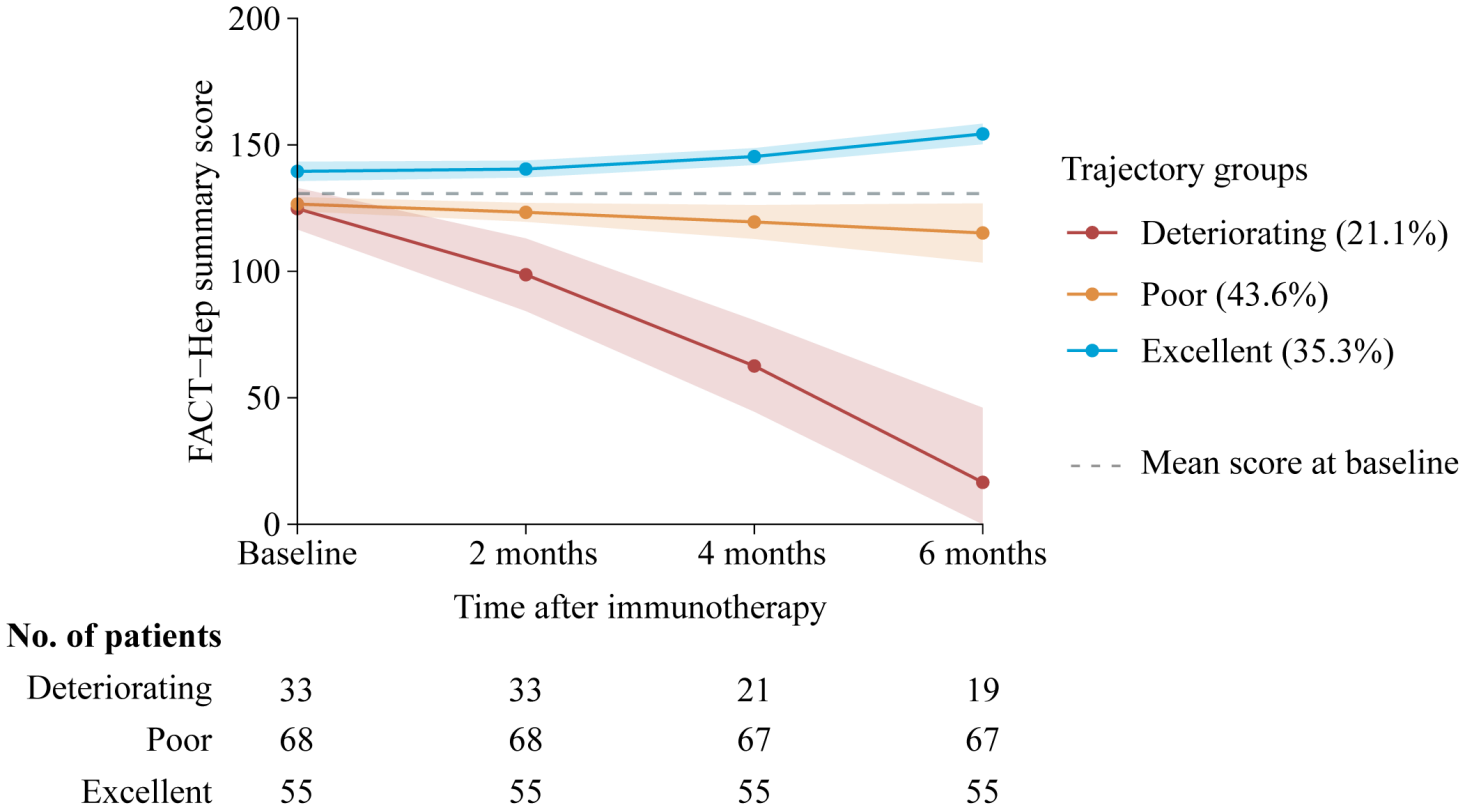
Trajectory groups according to best-fitting model (*n*=156). Solid lines represent the predicted trajectories, shadow shapes represent the respective 95% CIs, and dotted gray lines represent the mean score at basleline. FACT-Hep Summary Scores were available for 156 patients at baseline, and then among 156 at 2 months follow-up; 143 at 4 months follow-up; and 141 patients at 6 months follow-up. Higher scores reflect better QOL. FACT-Hep, Functional Assessment of Cancer Therapy - Hepatobiliary questionnaire; QOL, quality of life.

**Figure 3:**
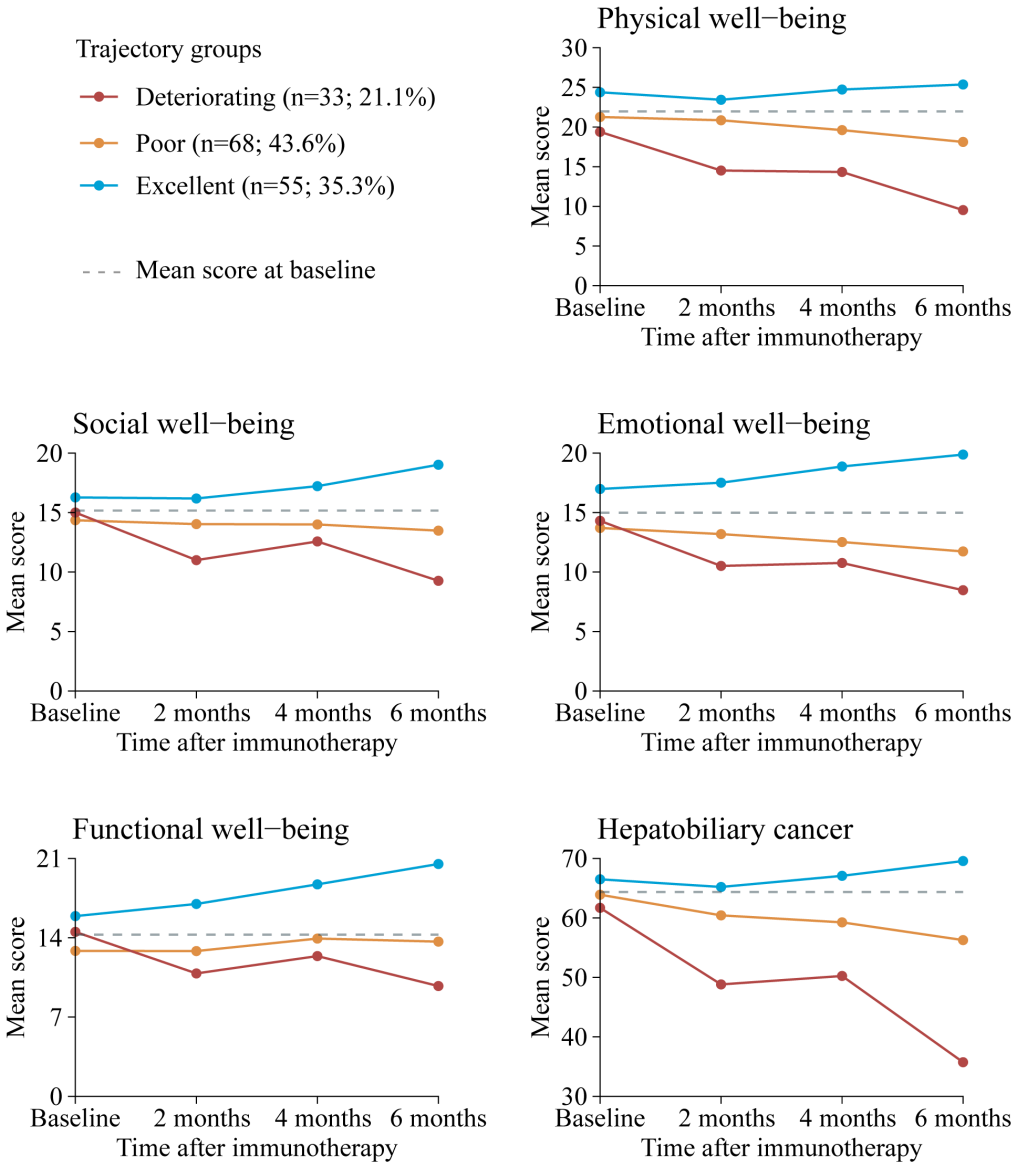
Mean QOL scores by trajectory group and by time point for FACT-Hep subscales. Solid lines represent the predicted trajectories, dotted gray lines represent the mean score at basleline. Higher scores indicate greater functionality. Respective 95% CIs for the means are available in **Supplementary Table S5**. FACT-Hep, Functional Assessment of Cancer Therapy - Hepatobiliary questionnaire; QOL, quality of life.

Our final model identified three trajectory groups (**Figure 2**). Model selection metrics are presented in **Supplementary Tables S2, S3**. The first trajectory group (n=55, 35.3%; excellent) demonstrated consistently QOL over time, with an estimated QOL score of 139.52 (95% CI, 135.71 to 143.33) at baseline and 154.32 (95% CI, 150.26 to 158.39) at month 6. The second trajectory group included the majority of patients (n=68, 43.6%; poor), who reported a consistently low QOL from baseline, with an estimated QOL score of 126.60 (95% CI, 123.83 to 129.36), declining to 115.16 (95% CI, 103.47 to 126.86) at month 6. In the third trajectory group (n=33, 21.1%; deteriorating), QOL at baseline was comparable to that of the second group, with a mean QOL score of 124.79 (95% CI, 116.58 to 133.00). However, a significant decline occurred by month 2, with an estimated QOL score of 98.67 (95% CI, 84.33 to 113.00), and the lowest mean score was recorded at month 6, at 16.58 (95% CI, 0 to 46.07). The detail estimated score and respective 95% CIs for the trajectory groups are available in the **Supplementary Table S4**. The trend of five subscalew of QOL in different trajectories was basically the same (**Figure 3**).

### 3.3 Trajectory group membership

**Table 1** displays patient characteristics by trajectory group. Univariabel analysis (**Supplementary Table S6**) revealed that sex, drinking status, diabetes, body mass index, extrahepatic metastasis, vascular invasion, BCLC stage, transarterial chemoembolization, and radiotherapy may be the trajectory group membership. We incorporated these factors into multivariable multinomial logistic regression model (**Table 2**) for further validation, we excluded sex, due to the sample size of women is very small. Patients with no alcohol consumption were significantly associated with poor and deteriorating trajectory groups, with OR of 5.55 (95% CI: 2.44–12.50) and 3.70 (95% CI: 1.28–11.11), respectively. Similarly, those who did not receive radiotherapy had ORs of 5.00 (95% CI: 1.54–16.67) for poor and 8.33 (95% CI: 1.41–50.00) for deteriorating outcomes. Patients with a BMI ≤24.0 kg/m² were more likely to be in poor QOL trajectory groups (OR: 4.49; 95% CI: 1.65–12.22). Additionally, patients with diabetes and extrahepatic metastasis had ORs of 6.83 (95% CI: 1.57–29.73) and 3.08 (95% CI: 1.07–8.87), respectively, indicating higher likelihoods of deteriorating QOL patterns compared to those with excellent trajectories.

**Table 1:** Distribution of patient characteristics at baseline by quality of life trajectory group (n=156).

Characteristic	Excellent (n=55)	Poor (n=68)	Deteriorating (n=33)	P value ^a^
Age, years				
Mean ± SD	52.20 ± 10.18	50.25 ± 11.28	49.67 ± 9.23	0.462
<60	43 (78.2%)	52 (76.5%)	30 (90.9%)	0.211
≥60	12 (21.8%)	16 (23.5%)	3 (9.1%)	
Sex				0.001
Male	53 (96.4%)	54 (79.4%)	33 (100.0%)	
Female	2 (3.6%)	14 (20.6%)	0 (0.0%)	
Drinking status				<0.001
No	20 (36.4%)	49 (72.1%)	20 (60.6%)	
Yes	35 (63.6%)	19 (27.9%)	13 (39.4%)	
Monthly household income, yuan				0.800
<6000	44 (80.0%)	57 (83.8%)	28 (84.8%)	
≥6000	11 (20.0%)	11 (16.2%)	5 (15.2%)	
Family history of cancer				0.957
No	43 (78.2%)	52 (76.5%)	26 (78.8%)	
Yes	12 (21.8%)	16 (23.5%)	7 (21.2%)	
Diabetes				0.008
No	49 (89.1%)	64 (94.1%)	24 (72.7%)	
Yes	6 (10.9%)	4 (5.9%)	9 (27.3%)	
Hypertension				0.344
No	41 (74.5%)	57 (83.8%)	28 (84.8%)	
Yes	14 (25.5%)	11 (16.2%)	5 (15.2%)	
Hepatitis B surface antigen				0.228
Negative	5 (9.1%)	5 (7.4%)	6 (18.2%)	
Positive	50 (90.9%)	63 (92.6%)	27 (81.8%)	
Hepatitis C antibody				0.053
Negative	51 (92.7%)	68 (100.0%)	32 (97.0%)	
Positive	4 (7.3%)	0 (0.0%)	1 (3.0%)	
Liver cirrhosis				0.844
No	16 (29.1%)	23 (33.8%)	10 (30.3%)	
Yes	39 (70.9%)	45 (66.2%)	23 (69.7%)	
Body mass index, kg/m^2^				
Mean ± SD	23.02 ± 3.51	21.55 ± 2.55	21.65 ± 3.21	0.022
≤24.0	36 (65.5%)	58 (85.3%)	27 (81.8%)	0.026
>24.0	19 (34.5%)	10 (14.7%)	6 (18.2%)	
Tumor number				0.996
Single	10 (18.2%)	12 (17.6%)	6 (18.2%)	
Multiple	45 (81.8%)	56 (82.4%)	27 (81.8%)	
Tumor size, cm				0.452
<5	12 (21.8%)	9 (13.2%)	6 (18.2%)	
≥5	43 (78.2%)	59 (86.8%)	27 (81.8%)	
Extrahepatic metastasis				0.001
No	39 (70.9%)	51 (75.0%)	13 (39.4%)	
Yes	16 (29.1%)	17 (25.0%)	20 (60.6%)	
Vascular invasion				0.096
No	20 (36.4%)	22 (32.4%)	5 (15.2%)	
Yes	35 (63.6%)	46 (67.6%)	28 (84.8%)	
a-Fetoprotein, ng/ml				0.664
≤400	27 (49.1%)	32 (47.1%)	13 (39.4%)	
>400	28 (50.9%)	36 (52.9%)	20 (60.6%)	
BCLC stage				0.134
B	17 (30.9%)	16 (23.5%)	4 (12.1%)	
C	38 (69.1%)	52 (76.5%)	29 (87.9%)	
Targeted therapy regimens				0.807
Donafenib	9 (16.4%)	13 (19.1%)	5 (15.2%)	
Lenvatinib	35 (63.6%)	47 (69.1%)	24 (72.7%)	
Regorafenib	0 (0.0%)	1 (1.5%)	0 (0.0%)	
Apatinib	2 (3.6%)	3 (4.4%)	2 (6.1%)	
Anrotinib	1 (1.8%)	0 (0.0%)	0 (0.0%)	
Bevacizumab	8 (14.5%)	4 (5.9%)	2 (6.1%)	
Immunotherapy regimens				0.585
Atezolizumab	2 (3.6%)	1 (1.5%)	0 (0.0%)	
Cetuximab	7 (12.7%)	3 (4.4%)	3 (9.1%)	
Camrelizumab	10 (18.2%)	12 (17.6%)	8 (24.2%)	
Tislelizumab	36 (65.5%)	48 (70.6%)	21 (63.6%)	
Penpulimab	0 (0.0%)	2 (2.9%)	1 (3.0%)	
Pembrolizumab	0 (0.0%)	2 (2.9%)	0 (0.0%)	
Liver resection				0.787
No	49 (89.1%)	58 (85.3%)	28 (84.8%)	
Yes	6 (10.9%)	10 (14.7%)	5 (15.2%)	
Transarterial chemoembolization				0.033
No	7 (12.7%)	10 (14.7%)	11 (33.3%)	
Yes	48 (87.3%)	58 (85.3%)	22 (66.7%)	
Radiotherapy				0.066
No	43 (78.2%)	61 (89.7%)	31 (93.9%)	
Yes	12 (21.8%)	7 (10.3%)	2 (6.1%)	

Data are presented as n(%), unless otherwise indicated.

BCLC, Barcelona clinic liver cancer; SD, standard deviation.

a P value was based on χ^2^, t-test where appropriate.

**Table 2:** Multinomial logistic regression of factors associated with FACT-Hep score trajectory group membership (*vs* reference Excellent).

Factors	Poor (n=68)		Deteriorating (n=55)	
	OR (95% CI)	P value	OR (95% CI)	P value
Drinking status				
Yes	Reference		Reference	
No	5.55 (2.44–12.50)	<0.001	3.70 (1.28–11.11)	0.016
Diabetes				
No	Reference		Reference	
Yes	0.65 (0.15–2.78)	0.566	6.83 (1.57–29.73)	0.010
Body mass index, kg/m^2^				
>24.0	Reference		Reference	
≤24.0	4.49 (1.65–12.22)	0.003	3.22 (0.91–11.42)	0.071
Extrahepatic metastasis				
No	Reference		Reference	
Yes	0.70 (0.27–1.79)	0.458	3.08 (1.07–8.87)	0.037
Vascular invasion				
No	Reference		Reference	
Yes	0.69 (0.25–1.87)	0.463	2.48 (0.60–10.20)	0.208
BCLC stage				
B	Reference		Reference	
C	2.15 (0.72–6.45)	0.173	2.21 (0.49–9.94)	0.299
Transarterial chemoembolization				
No	Reference		Reference	
Yes	0.73 (0.21–2.49)	0.617	0.29 (0.08–1.08)	0.064
Radiotherapy				
Yes	Reference		Reference	
No	5.00 (1.54–16.67)	0.008	8.33 (1.41–50.00)	0.019

OR, odds ratio; CI, confidence interval.

## 4 Discussion

This study aimed to characterize the QOL trajectories among patients with intermediate and advanced HCC treated with immunotherapy. Through the use of group-based trajectory modeling, we identified three distinct QOL trajectories: excellent, poor, and deteriorating. Our findings indicate that a significant portion of patients experienced severe and sustained declines in QOL, with the lowest scores observed at the six-month mark. Factors such as no alcohol consumption, absence of radiotherapy, diabetes, and extrahepatic metastasis were significantly associated with membership in the deteriorating group. These results highlight the necessity for targeted interventions to mitigate the adverse impacts of immunotherapy on patient QOL.

QOL in HCC patients is influenced by medical factors, including the disease itself, its complications, treatments such as oncological and immunotherapy, underlying liver conditions, and psychological, social, or spiritual responses. One interesting use of QOL data in HCC patients is prognostication for OS. A study identified worse scores in appetite loss, physical function, and role function from the EORTC QLQ-C30 as independent risk factors for shorter OS in advanced HCC patients (26). Another study using the EORTC QLQ-C30 found that a better baseline role function score was a significant prognostic factor for longer OS in advanced HCC patients (27). The baseline Spitzer QoL index was reported as prognostic for survival in 538 advanced HCC patients, with higher scores associated with longer OS (28). Attempts have been made to enhance existing staging systems with QOL data (27, 28). Addition of EORTC QLQ-C30 data has been shown to improve the performance of the Cancer of the Liver Italian Program (CLIP) (29), the BCLC system (30), the Groupe d’Étude et de Traitement du Carcinome Hépatocellulaire system (31). Spitzer QOL index could improve the prognostic value of CLIP (28). In addition, important utilization of QOL in HCC patients included description of symptomatology and QOL of patients, treatment endpoint in clinical trial, and health care valuation (17, 32). QOL measurement provides valuable information in clinical practice and research. Future research into utilization in clinical trials as well as routine clinical practice are warranted. While novel immunotherapies have improved overall survival in HCC patients in recent years, their unique side effects may reduce overall treatment efficacy and perceived benefits (33). Therefore, regular monitoring of changes in quality of life can provide important clues to the long-term prognosis of patients. Previous studies have shown that immunotherapy often has a transient negative effect on QOL (19, 20). However, traditional analytic methods, which describe average outcomes at the population level, may fail to capture the individual variability in the longitudinal trajectory of cancer-related QOL. Therefore, this study used latent categorical analysis to identify subgroups of patients with similar longitudinal trajectories, thereby providing a more comprehensive picture of the quality of life of HCC patients after immunotherapy and its impact over time.

In our cohort, the deteriorating trajectory group exhibited significant declines in QOL as early as three weeks post-treatment, with the lowest scores at six months. This rapid deterioration underscores the severe impact of immunotherapy on patients’ well-being and highlights the necessity for early intervention. Our findings contribute to the growing body of literature on the short-term effects of cancer treatments on patient-reported outcomes. While previous studies have often focused on QOL at only one time point (19, 20), our research provides a comprehensive assessment of overall QOL trajectories over an extended period. This approach allows for a more nuanced understanding of the short-term impact of immunotherapy on HCC patients and underscores the need for continuous monitoring and intervention to support patients’ well-being throughout their treatment journey.

The identification of modifiable or non-modifiable factors, as predictors of deteriorating QOL trajectories underscores the importance of personalized care strategies. Patients who abstained from alcohol were more likely to be in the deteriorating QOL group (OR=3.70, 95% CI, 1.28–11.11). Moderate alcohol consumption is often associated with positive social interactions and emotional support. It serves as a part of social activities, enhancing social connections which can positively impact mental health (34, 35). However, the relationship between alcohol consumption and QOL requires further study, as it varies individually and is influenced by factors such as quantity consumed, drinking patterns, and personal health status (36). Patients who did not receive radiotherapy were more likely to be in the deteriorating QOL group (OR=8.33, 95% CI, 1.41–50.00). Radiotherapy plays a significant role in controlling tumor growth and alleviating symptoms, and enhances immunotherapeutic sensitivity (37, 38). Patients who do not undergo radiotherapy may experience more pain and other symptoms due to inadequate tumor control, directly lowering their QOL (39). Additionally, patients receiving radiotherapy often have higher expectations and confidence in treatment outcomes, which positively influences QOL (39). Although radiotherapy may have some side effects, the benefits in symptom relief and QOL improvement generally outweigh the negatives. Patients with a low BMI (≤24.0 kg/m²) were more likely to be in the poor QOL group (OR=4.49, 95% CI, 1.65–12.22). A low BMI often indicates malnutrition or insufficient body weight, leading to compromised immune function, physical decline, and worsening health conditions, thereby impacting QOL (40, 41). Moreover, a low BMI could signify underlying conditions such as cachexia, a complex metabolic syndrome further reducing patient QOL (42). Patients with diabetes were more likely to be in the deteriorating QOL group (OR=6.83, 95% CI, 1.57–29.73). Diabetes commonly accompanies multiple complications such as cardiovascular disease, kidney disease, neuropathy, and retinopathy (43, 44), significantly affecting both health and QOL (45, 46). Managing diabetes requires long-term medication and strict dietary control, placing high demands on patients’ lifestyle and mental state, thereby increasing their burden of living (47). Additionally, diabetes patients often experience psychological distress such as anxiety and depression (48), further impacting QOL. Patients with extrahepatic metastasis were more likely to be in the deteriorating QOL group (OR=3.08, 95% CI, 1.07–8.87). Extrahepatic metastasis typically indicates advanced disease and poor prognosis, significantly impacting patient QOL (49–51). It signifies tumor spread beyond the liver, accompanied by more severe symptoms and worse prognosis. These patients often require complex and invasive treatments, which themselves may negatively affect QOL (52).

In summary, the results of this study indicate that abstaining from alcohol, not receiving radiotherapy, low BMI, diabetes, and extrahepatic metastasis are critical factors influencing patient QOL. These factors collectively contribute to a significant decline in QOL among patients in the deteriorating group. Recognizing and intervening in these high-risk factors is crucial in clinical practice to improve patient QOL. For instance, providing nutritional support for malnourished patients (53), comprehensive management for diabetes patients (54), and considering radiotherapy (55) when appropriate may help enhance patient QOL. Additionally, offering psychological support and social resources could also positively impact the QOL of these patients (56). Future research should further explore the causal relationships between these factors and QOL, as well as assess the effectiveness of different interventions. This will provide more scientific and comprehensive guidance for clinical practice. Through integrated management and personalized treatments, it is possible to improve patient QOL and enhance their overall health outcomes.

From a clinical perspective, our findings are particularly relevant for informing patient care strategies. Greater treatment-related symptom burden is among the main reasons for non-adherence and discontinuation of treatment, which ultimately can contribute to poorer clinical outcomes. In the context of immunotherapy, it is crucial to identify patients at risk of significant QOL deterioration early in the treatment process. By doing so, healthcare providers can implement timely interventions to manage symptoms and support patients, potentially improving adherence to treatment and overall outcomes.

The strengths of our study include its prospective, longitudinal design and the use of a robust higher-order QOL outcome measure that summarizes multiple scales into a multidimensional response profile. This approach avoids the limitations of multiple comparisons and offers a comprehensive view of patients’ QOL over time. Moreover, the use of group-based trajectory modeling allows for the identification of clinically relevant latent groups, providing valuable insights into the diverse experiences of HCC patients undergoing immunotherapy. We acknowledge several limitations in this study. First, being a longitudinal investigation, it is subject to the common constraint of escalating response attrition as time progresses from study entry. Consequently, we recognize the potential for selection and attrition bias evolving over time. However, it is noteworthy that GBTM can effectively accommodate missing outcome data (25). Second, there are some well-known determinants of QOL that could not be explored, including psychologic measures such as depression and fatigue (57). Third, our models describe a population of patients with intermediate and advanced HCC, and the findings may not be generalizable to patients with early-stage disease. Further research is needed to confirm our findings in larger and more diverse patient populations and to explore the long-term impact of immunotherapy on QOL beyond the six-month period examined in this study. Fourth, our study population exclusively comprised Chinese survivors, limiting the generalizability of results. Fourth, we fitted the latent class model with a smaller sample size. Although our sample size may be relatively modest for latent class modeling, but our model evaluation metrics support the suitability of the fitted latent trajectory model. Finally, it is pertinent to note that the risk models for membership in specific trajectory groups may underestimate the uncertainty inherent in the trajectory modeling during the initial stage.

## 5 Conclusions

Our study identifies a high-risk cluster of HCC patients with severe, persistent QOL deterioration following immunotherapy. Screening for relevant patient-level characteristics can inform tailored interventions to mitigate the detrimental impact of immunotherapy and preserve QOL. Future research should focus on developing and testing targeted interventions that address both modifiable and non-modifiable factors associated with QOL declines in this patient population. This will be crucial in improving long-term outcomes and enhancing the overall quality of life for HCC patients undergoing immunotherapy.

## Data Availability

The data analyzed in this study is subject to the following licenses/restrictions: Data available on request due to privacy/ethical restrictions. Requests to access these datasets should be directed to Rong-Rui Huo, huorongrui@sr.gxmu.edu.cn.
