# Supplementary material for: Dynamics trajectory of patient-reported quality of life and its associated risk factors among hepatocellular carcinoma patients receiving immune checkpoint inhibitors: a prospective cohort study
Source: Front Immunol. 2024 Nov 4;15:1463655. doi: 10.3389/fimmu.2024.1463655 (PMC11570585; doi:10.3389/fimmu.2024.1463655)
Supplement: Supplementary file 1 [file DataSheet1.docx]

**Supplementary**

**Dynamics trajectory of patient-reported quality of life and its associated risk factors among hepatocellular carcinoma patients receiving immune checkpoint inhibitors: a prospective cohort study**

Xue-Mei You, Fei-Chen Lu, Fan-Rong Li, Feng-Juan Zhao, Rong-Rui Huo

Content

[Supplementary Methods 2](#_Toc170332232)

[Definition of trajectory groups 2](#_Toc170332233)

[Supplementary Tables 4](#_Toc170332234)

[Table S1. Baseline patient characteristics in the overall analytic cohort (n=156) 4](#_Toc170332235)

[Table S2. Metrics used for comparison across tested trajectory group models. 7](#_Toc170332236)

[Table S3. Maximum likelihood estimates of the model parameters 8](#_Toc170332237)

[Table S4. Mean score values and respective 95% CIs by trajectory-group 9](#_Toc170332238)

[Table S5. Mean scores and respective SD for all scales included in the FACT−Hep 10](#_Toc170332239)

[Table S6. Univariable analysis of factors associated with FACT-Hep score trajectory group membership (*vs* reference Excellent group) 11](#_Toc170332240)

# Supplementary Methods

## Definition of trajectory groups

Longitudinal variations in the repeated measures of the FACT-Hep Summary Score were evaluated using multivariable latent-class models via Group-Based Trajectory Modeling (GBTM). GBTM delineates the trajectory of outcomes over time by segmenting data into groups and identifying latent clusters of individuals following similar courses of the defined outcome^[1-3]^. Model selection involved iterative estimation to determine the optimal number and shape of trajectory groups using maximum likelihood methods.

We started with a single-group model and increased groups until identifying the best fit, adjusting polynomial terms based on significance and employing stepwise elimination of nonsignificant terms. The final model required all retained polynomial terms within each trajectory group to be statistically significant at the alpha = 0.05 level. Selection was based on the model with the smallest Sample Bayesian Information Criterion (SBIC) value.

Following model selection, we assessed model adequacy by examining the proportions of the sample assigned to each identified trajectory group. This assessment utilized estimated probabilities of group membership, posterior group probabilities (PP), Odds of Correct Classification (OCC), and the proportion assigned to each trajectory group based on average PP (AvePP). Criteria for confirming the selected model included achieving close correspondence between estimated and AvePP-based probabilities of group membership, AvePP values exceeding 0.70 for individuals in each trajectory group, and OCC values exceeding 5.

Subjective, non-statistical criteria were then applied to evaluate the final model selection, ensuring reasonable sample sizes within each trajectory group and non-overlapping, tight confidence intervals around estimated outcome evolution values. These subjective judgments were crucial in selecting the optimal trajectory model, particularly in cases where model fit criteria favored different models. Ultimately, the goal was to identify a final model that is not only statistically robust but also parsimonious and clinically relevant^[1-3]^.

**References**

[1] Nagin DS, Odgers CL. Group-based trajectory modeling in clinical research. Annu Rev Clin Psychol. 2010;6:109-138. doi: 10.1146/annurev.clinpsy.121208.131413.

[2] Nagin DS, Jones BL, Passos VL, Tremblay RE. Group-based multi-trajectory modeling. Stat Methods Med Res. 2018;27(7):2015-2023. doi: 10.1177/0962280216673085.

[3] Choi CW, Stone RA, Kim KH, Ren D, Schulz R, Given CW, et al. Group-based trajectory modeling of caregiver psychological distress over time. Ann Behav Med. 2012;44(1):73-84. doi: 10.1007/s12160-012-9371-8.

# Supplementary Tables

## Table S1. Baseline patient characteristics in the overall analytic cohort (n=156)

| Characteristic | n (%) |
| --- | --- |
| Age, years |  |
| Mean±SD | 50.81±10.48 |
| <60 | 125 (80.1%) |
| ≥60 | 31 (19.9%) |
| Sex |  |
| Male | 140 (89.7%) |
| Female | 16 (10.3%) |
| Drinking status |  |
| No | 89 (57.1%) |
| Yes | 67 (42.9%) |
| Monthly household income, yuan |  |
| <6000 | 129 (82.7%) |
| ≥6000 | 27 (17.3%) |
| Family history of cancer |  |
| No | 121 (77.6%) |
| Yes | 35 (22.4%) |
| Diabetes |  |
| No | 137 (87.8%) |
| Yes | 19 (12.2%) |
| Hypertension |  |
| No | 126 (80.8%) |
| Yes | 30 (19.2%) |
| Hepatitis B surface antigen |  |
| Negative | 16 (10.3%) |
| Positive | 140 (89.7%) |
| Hepatitis C antibody |  |
| Negative | 151 (96.8%) |
| Positive | 5 (3.2%) |
| Liver cirrhosis |  |
| No | 49 (31.4%) |
| Yes | 107 (68.6%) |
| Body mass index, kg/m^2^ |  |
| Mean±SD | 22.09±3.12 |
| ≤24.0 | 121 (77.6%) |
| >24.0 | 35 (22.4%) |
| Tumor number |  |
| Single | 28 (17.9%) |
| Multiple | 128 (82.1%) |
| Tumor size, cm |  |
| <5 | 27 (17.3%) |
| ≥5 | 129 (82.7%) |
| Extrahepatic metastasis |  |
| No | 103 (66.0%) |
| Yes | 53 (34.0%) |
| Vascular invasion |  |
| No | 47 (30.1%) |
| Yes | 109 (69.9%) |
| a-Fetoprotein, ng/ml |  |
| ≤400 | 72 (46.2%) |
| >400 | 84 (53.8%) |
| BCLC stage |  |
| B | 37 (23.7%) |
| C | 119 (76.3%) |
| Targeted therapy regimens |  |
| Donafenib | 27 (17.3%) |
| Lenvatinib | 106 (67.9%) |
| Regorafenib | 1 (0.6%) |
| Apatinib | 7 (4.5%) |
| Anrotinib | 1 (0.6%) |
| Bevacizumab | 14 (9.0%) |
| Immunotherapy regimens |  |
| Atezolizumab | 3 (1.9%) |
| Cetuximab | 13 (8.3%) |
| Camrelizumab | 30 (19.2%) |
| Tislelizumab | 105 (67.3%) |
| Penpulimab | 3 (1.9%) |
| Pembrolizumab | 2 (1.3%) |
| Liver resection |  |
| No | 135 (86.5%) |
| Yes | 21 (13.5%) |
| Transarterial chemoembolization |  |
| No | 28 (17.9%) |
| Yes | 128 (82.1%) |
| Radiotherapy |  |
| No | 135 (86.5%) |
| Yes | 21 (13.5%) |

Data are presented as n(%), unless otherwise indicated.

Abbreviations: BCLC, Barcelona clinic liver cancer; SD: standard deviation.

## Table S2. Metrics used for comparison across tested trajectory group models.

| Groups | SBIC | Relative entropy | Estimated probability  of group membership | AvePP ^a^ | OCC |
| --- | --- | --- | --- | --- | --- |
| **Group = 2** | 4924.995 | 0.828 |  |  |  |
| Class 1 |  |  | 21.1% | 0.941 | 54.452 |
| Class 2 |  |  | 78.8% | 0.965 | 7.913 |
| **Group = 3** | 4858.814 | 0.818 |  |  |  |
| Class 1 |  |  | 21.1% | 0.905 | 33.722 |
| Class 2 |  |  | 35.3% | 0.847 | 11.702 |
| Class 3 |  |  | 43.6% | 0.895 | 70.026 |
| **Group = 4** | 4861.858 | 0.881 |  |  |  |
| Class 1 |  |  | 14.1% | 0.959 | 141.959 |
| Class 2 |  |  | 32.7% | 0.925 | 24.493 |
| Class 3 |  |  | 46.8% | 0.949 | 21.586 |
| Class 4 |  |  | 6.4% | 0.918 | 170.719 |
| **Group = 5** | 4874.739 | 0.716 |  |  |  |
| Class 1 |  |  | 16.0% | 0.892 | 37.940 |
| Class 2 |  |  | 21.8% | 0.741 | 11.581 |
| Class 3 |  |  | 19.9% | 0.738 | 9.493 |
| Class 4 |  |  | 36.5% | 0.874 | 13.657 |
| Class 5 |  |  | 5.8% | 0.918 | 186.693 |

Abbreviations: SBIC, Sample bayesian information criterion; AvePP, Average posterior probability; OCC, Odds of correct classification.

^a^ An average posterior probability of group membership close to or >0.80 indicates a relatively high degree of certainty in group assignment.

## Table S3. Maximum likelihood estimates of the model parameters

| Term | B | S.E. | Wlad χ^2^ | P value |
| --- | --- | --- | --- | --- |
| Deteriorating (n=33; 21.1%) |  |  |  |  |
| Intercept | 140.979 | 8.898 | 15.843 | <0.001 |
| Linear | -11.213 | 7.704 | -1.455 | 0.146 |
| Quadratic | -4.972 | 1.101 | -4.516 | <0.001 |
| Excellent (n=55; 35.3%) |  |  |  |  |
| Intercept | 142.614 | 3.677 | 38.788 | <0.001 |
| Linear | -5.099 | 2.993 | -1.704 | 0.088 |
| Quadratic | 2.006 | 0.595 | 3.372 | 0.001 |
| Poor (n=68; 43.6%) |  |  |  |  |
| Intercept | 129.307 | 3.270 | 39.541 | <0.001 |
| Linear | -2.437 | 3.031 | -0.804 | 0.421 |
| Quadratic | -0.275 | 0.737 | -0.373 | 0.709 |

Trajectories of FACT-Hep score for groups “Deteriorating”, and “Excellent” are described with an intercept plus a quadratic (P<0.001 for the two groups) time parameter. The “Poor” group is further described with an intercept (P<0.001).

## Table S4. Mean score values and respective 95% CIs by trajectory-group

| Time after immunotherapy | Deteriorating  (n=33; 21.1%) | Poor  (n=68; 43.6%) | Excellent  (n=55; 35.3%) |
| --- | --- | --- | --- |
| Baseline | 124.79 (116.58 to 133.00) | 126.60 (123.83 to 129.36) | 139.52 (135.71 to 143.33) |
| 2 months | 98.67 (84.33 to 113.00) | 123.33 (119.63 to 127.04) | 140.44 (137.11 to 143.77) |
| 4 months | 62.60 (44.54 to 80.65) | 119.52 (112.82 to 126.22) | 145.38 (142.05 to 148.70) |
| 6 months | 16.58 (0 to 46.07) | 115.16 (103.47 to 126.86) | 154.32 (150.26 to 158.39) |

## Table S5. Mean scores and respective SD for all scales included in the FACT−Hep

| Domain | Excellent (n=55) | Poor (n=68) | Deteriorating (n=33) | P value |
| --- | --- | --- | --- | --- |
| **At baseline (n=156)** |  |  |  |  |
| Physical well-being | 24.38±3.17 | 21.26±4.08 | 19.39±5.36 | <0.001 |
| Social well-being | 16.27±2.73 | 14.35±2.46 | 15.00±3.54 | 0.001 |
| Emotional well-being | 16.98±2.39 | 13.71±2.50 | 14.30±3.72 | <0.001 |
| Functional well-being | 15.91±3.93 | 12.84±3.41 | 14.52±3.32 | <0.001 |
| Hepatobiliary cancer | 66.49±4.54 | 63.90±5.91 | 61.70±11.03 | 0.006 |
| **Three weeks after immunotherapy (n=156)** | |  |  |  |
| Physical well-being | 23.44±3.30 | 20.85±3.88 | 14.52±7.03 | <0.001 |
| Social well-being | 16.18±2.63 | 14.03±2.59 | 11.00±4.23 | <0.001 |
| Emotional well-being | 17.51±2.19 | 13.19±3.20 | 10.52±4.49 | <0.001 |
| Functional well-being | 16.98±5.01 | 12.82±3.97 | 10.85±4.72 | <0.001 |
| Hepatobiliary cancer | 65.20±3.11 | 60.43±5.31 | 48.82±14.48 | <0.001 |
| **Six weeks after immunotherapy (n=143)** | |  |  |  |
| Physical well-being | 24.73±2.93 | 19.61±4.39 | 14.33±5.89 | <0.001 |
| Social well-being | 17.22±2.55 | 14.00±2.82 | 12.57±4.25 | <0.001 |
| Emotional well-being | 18.87±1.69 | 12.52±3.44 | 10.76±4.44 | <0.001 |
| Functional well-being | 18.71±3.66 | 13.93±4.50 | 12.38±4.93 | <0.001 |
| Hepatobiliary cancer | 67.07±2.84 | 59.25±7.52 | 50.24±15.15 | <0.001 |
| **Six months after immunotherapy (n=141)** | |  |  |  |
| Physical well-being | 25.36±2.89 | 18.12±5.27 | 9.53±4.71 | <0.001 |
| Social well-being | 19.02±2.28 | 13.48±3.40 | 9.26±3.96 | <0.001 |
| Emotional well-being | 19.87±1.96 | 11.73±3.77 | 8.47±3.85 | <0.001 |
| Functional well-being | 20.51±3.20 | 13.66±4.35 | 9.74±5.08 | <0.001 |
| Hepatobiliary cancer | 69.56±2.86 | 56.27±9.49 | 35.74±14.93 | <0.001 |

## Table S6. Univariable analysis of factors associated with FACT-Hep score trajectory group membership (*vs* reference Excellent group)

| Factors | Poor (n=68) | |  | Deteriorating (n=33) | |
| --- | --- | --- | --- | --- | --- |
|  | OR (95% CI) | P value |  | OR (95% CI) | P value |
| Age, years |  |  |  |  |  |
| <60 | Reference |  |  | Reference |  |
| ≥60 | 1.10 (0.47–2.58) | 0.822 |  | 0.36 (0.09–1.38) | 0.136 |
| Sex |  |  |  |  |  |
| Male | Reference |  |  | Reference |  |
| Female | 6.87 (1.49–31.73) | 0.014 |  | NA | NA |
| Drinking status |  |  |  |  |  |
| No | Reference |  |  | Reference |  |
| Yes | 0.22 (0.10–0.48) | <0.001 |  | 0.37 (0.15–0.90) | 0.029 |
| Monthly household income, yuan |  |  |  |  |  |
| <6000 | Reference |  |  | Reference |  |
| ≥6000 | 0.77 (0.31–1.94) | 0.583 |  | 0.71 (0.22–2.27) | 0.569 |
| Family history of cancer |  |  |  |  |  |
| No | Reference |  |  | Reference |  |
| Yes | 1.10 (0.47–2.58) | 0.822 |  | 0.97 (0.34–2.76) | 0.948 |
| Diabetes |  |  |  |  |  |
| No | Reference |  |  | Reference |  |
| Yes | 0.51 (0.14–1.91) | 0.318 |  | 3.06 (0.98–9.60) | 0.055 |
| Hypertension |  |  |  |  |  |
| No | Reference |  |  | Reference |  |
| Yes | 0.57 (0.23–1.37) | 0.207 |  | 0.52 (0.17–1.62) | 0.260 |
| Hepatitis B surface antigen |  |  |  |  |  |
| Negative | Reference |  |  | Reference |  |
| Positive | 1.26 (0.35–4.60) | 0.726 |  | 0.45 (0.13–1.61) | 0.220 |
| Hepatitis C antibody |  |  |  |  |  |
| Negative | Reference |  |  | Reference |  |
| Positive | NA | NA |  | 0.40 (0.04–3.72) | 0.418 |
| Liver cirrhosis |  |  |  |  |  |
| No | Reference |  |  | Reference |  |
| Yes | 0.80 (0.37–1.73) | 0.575 |  | 0.94 (0.37–2.42) | 0.904 |
| Body mass index, kg/m^2^ |  |  |  |  |  |
| ≤24.0 | Reference |  |  | Reference |  |
| >24.0 | 0.33 (0.14–0.78) | 0.012 |  | 0.42 (0.15–1.20) | 0.105 |
| Tumor number |  |  |  |  |  |
| Single | Reference |  |  | Reference |  |
| Multiple | 1.04 (0.41–2.62) | 0.939 |  | 1.00 (0.33–3.06) | >0.999 |
| Tumor size, cm |  |  |  |  |  |
| <5 | Reference |  |  | Reference |  |
| ≥5 | 1.83 (0.71–4.73) | 0.212 |  | 1.26 (0.42–3.74) | 0.683 |
| Extrahepatic metastasis |  |  |  |  |  |
| No | Reference |  |  | Reference |  |
| Yes | 0.81 (0.37–1.81) | 0.611 |  | 3.75 (1.51–9.31) | 0.004 |
| Vascular invasion |  |  |  |  |  |
| No | Reference |  |  | Reference |  |
| Yes | 1.19 (0.57–2.53) | 0.641 |  | 3.20 (1.07–9.60) | 0.038 |
| a-Fetoprotein, ng/ml |  |  |  |  |  |
| ≤400 | Reference |  |  | Reference |  |
| >400 | 1.08 (0.53–2.21) | 0.823 |  | 1.48 (0.62–3.56) | 0.377 |
| BCLC stage |  |  |  |  |  |
| B | Reference |  |  | Reference |  |
| C | 1.45 (0.65–3.24) | 0.360 |  | 3.24 (0.99–10.68) | 0.053 |
| Liver resection |  |  |  |  |  |
| No | Reference |  |  | Reference |  |
| Yes | 1.41 (0.48–4.15) | 0.535 |  | 1.46 (0.41–5.22) | 0.561 |
| Transarterial chemoembolization |  |  |  |  |  |
| No | Reference |  |  | Reference |  |
| Yes | 0.85 (0.30–2.39) | 0.752 |  | 0.29 (0.10–0.85) | 0.024 |
| Radiotherapy |  |  |  |  |  |
| No | Reference |  |  | Reference |  |
| Yes | 0.41 (0.15–1.13) | 0.085 |  | 0.23 (0.05–1.11) | 0.067 |

Abbreviations: BCLC, Barcelona clinic liver cancer; OR, odds ratio.
